# Growth of Co_2_FeAl Heusler alloy thin films on Si(100) having very small Gilbert damping by Ion beam sputtering

**DOI:** 10.1038/srep28692

**Published:** 2016-06-30

**Authors:** Sajid Husain, Serkan Akansel, Ankit Kumar, Peter Svedlindh, Sujeet Chaudhary

**Affiliations:** 1 Thin Film Laboratory, Department of Physics, Indian Institute of Technology Delhi, New Delhi 110016, India; 2 Ångström Laboratory, Department of Engineering Sciences, Box 534, SE-751 21 Uppsala, Sweden

## Abstract

The influence of growth temperature *T*_*s*_ (300–773 K) on the structural phase ordering, static and dynamic magnetization behaviour has been investigated in ion beam sputtered full Heusler alloy Co_2_FeAl (CFA) thin films on industrially important Si(100) substrate. The *B2* type magnetic ordering is established in these films based on the clear observation of the (200) diffraction peak. These ion beam sputtered CFA films possess very small surface roughness of the order of subatomic dimensions (<3 Å) as determined from the fitting of XRR spectra and also by AFM imaging. This is supported by the occurrence of distinct Kiessig fringes spanning over the whole scanning range (~4°) in the x-ray reflectivity (XRR) spectra. The Gilbert damping constant *α* and effective magnetization *4πM*_*eff*_ are found to vary from 0.0053 ± 0.0002 to 0.0015 ± 0.0001 and 13.45 ± 00.03 kG to 14.03 ± 0.04 kG, respectively. These Co_2_FeAl films possess saturation magnetization ranging from 4.82 ± 0.09 to 5.22 ± 0.10 *μ*_*B*_/*f.u*. consistent with the bulk *L2*_*1*_-type ordering. A record low *α*-value of 0.0015 is obtained for Co_2_FeAl films deposited on Si substrate at *T*_*s*_ ~ 573 K.

The interest in the Heusler alloy thin films is widely increasing due to properties like tunable anisotropy, large spin polarization and high Curie temperature (*T*_*c*_),making them attractive for spintronic applications; high density magnetic storage^1^,^2^, spin-transfer-torque magnetic random access memories^3^ and magnonic devices^4^. Conventional memory devices require strong thermal stability^5^ and low critical current density for spin transfer torque switching of the magnetization^6^. A low critical current density requires a magnetic material with a low Gilbert damping constant (*α*), which describes the rate of relaxation of the magnetization. However, as the ferromagnetic (FM) film thickness is reduced, it becomes difficult to maintain a low value of the damping constant^7^. Current research efforts include FM electrodes for spintronic devices with high spin polarization and low Gilbert damping constant. Very recently, Schoen *et al*. reported ultra-low damping in Co-Fe binary alloy thin films^8^. The Co-based Heusler alloys are also very promising spintronic materials owing to their high *T*_*c*_ (~1000 K) and 100% spin polarization due to the spin split band structure induced half-metallicity^9^. However, the anti-site disorder in these alloys significantly reduces *T*_*c*_ as well as the spin polarization^10^. The so-called full Heusler alloys, represented by X_2_YZ, e.g., Ni_2_MnSi, Co_2_MnSi, Co_2_MnGe, Co_2_FeAl, etc., exhibit three different types of atomically ordered phases, namely fully ordered *L2*_*1*_, partially ordered *B2* and fully disordered *A2* phases. In the *L2*_*1*_ phase, all the three types of atoms (X, Y and Z) occupy their respective assigned sites. However, in the *B2* phase, Y and Z atoms randomly share their positions, and the *A2* phase corresponds to a completely random occupation of all existing sites of the *L2*_*1*_ phase by any X, Y or Z atoms. The half-metallicity in Heusler alloys is sensitive to these atomic arrangements and degrades with atomic disorder, compared to the *L2*_*1*_ phase which is perfectly half-metallic. Picozzi *et al*. reported that the atomic disorder might lead to additional states at the Fermi level, thus reducing the spin polarization^9^,^10^. Among these alloys, Co_2_FeAl (CFA) is the most widely investigated Heusler alloy. Although several attempts have been made to grow fully ordered CFA thin films, especially on MgO substrate, using different growth techniques, the success in crystallizing the *L2*_*1*_ phase in Co_2_FeAl Heusler alloy thin film is far from being achieved. To our knowledge, only Qiao *et al*., reported *L2*_*1*_ films by MBE^11^.

In order to realize the fully ordered CFA phase, it is necessary to optimize the growth temperature (*T*_*s*_) and to use sufficiently high *T*_*s*_ so that on the one hand it promotes the formation of the *L2*_*1*_ phase while on the other hand it avoids atomic disorder which is a thermally activated process. Therefore, synthesis of highly ordered CFA thin films continues to be a challenging task. Very few attempts have made to grow CFA thin films on Si substrate^12^,^13^,^14^, a combination which is potentially advantageous for the silicon spintronics. In them, a limited success is reported with regards to achieving *B2* ordered CFA films^12^. For obtaining the ordered CFA phase, it is highly desirable that while the substrate is maintained at the optimum *T*_*s*_ the surface mobility of the ad-atom during the growth of the films is sufficiently high so that they can lower their energy by reaching to their respective atomic sites. In this regard, the ion beam sputtering technique^15^ offers great promises since in that the energy of the sputtered species can be controlled by varying the energy of the argon ion beam employed for sputtering, and the film growth takes place far away from the plasma at relatively lower pressure (~10^−5^ to 10^−4^ Torr range). This makes this technique an energy enhanced process in which sputtered species carry substantially large energy, ~20 eV, compared to other deposition techniques. This high energy of sputtered species is responsible for high ad-atom mobility of the growing films and reduction of the phase formation temperature. In this report, we present a detailed study on the structural and magnetic (static and dynamic) properties of CFA thin films grown on Si(100) substrate at different *T*_*s*_ by employing ion beam sputtering. The observed results demonstrate a direct correlation between the structure and dynamic response of the CFA thin films, tuned by optimizing the substrate temperature during the ion beam sputtering.

## Experiments

The CFA thin films of constant thickness were deposited on Si(100) at various temperatures using a ion-beam sputtering deposition system (NORDIKO-3450). Prior to the deposition, the Si(100) wafers were ultrasonically cleaned in acetone and propanol baths, and subsequently treated with HF (10:1 ratio) solution for 60s to remove the native SiO_2_ layer. Immediately after the treatment, substrates were loaded into the high vacuum (HV) chamber for deposition using a load-lock system. The substrates were then heat treated at 620 °C for 2 h for smoothening to achieve atomically flat substrate surfaces prior to film growth^16^. The HV chamber was evacuated down to ~7 × 10^−7^ Torr using a turbo pump. The *6*″ *dia*. CFA alloy target (composition Co_50_Fe_25_Al_25_) fixed on a remote controlled water cooled turret was sputtered by ~4.5 inch *dia*. high energy Ar-ion beam (500 eV) extracted from a RF ion-source (grid current/voltage parameters: V_+_ = 500V, I_+_ = 79 mA, V_−_ = −270V, and I_−_ = 5 mA). During the deposition, the chamber was maintained at ~2.4 × 10^−4^ Torr by bleeding 3.5 sccm Ar gas directly in to the ion-source operated at 75 W. A series of Si/CFA(53 nm)/Ta(2 nm) thin films were prepared by varying *T*_*s*_(300 K, 573 K, 673 K and 773 K) while keeping the other parameters constant (i.e., energy of Ar ions, Ar-flow rate and RF-power). A 2 nm thick layer of Ta was used as a capping layer to prevent the surface oxidation. It is to be noted that the above mentioned thicknesses are only the nominal thicknesses derived from measurements on thicker films using surface profilometry employing a Bruker *Dektak XT* profiler system (Billerica, MA, USA). The actual film thicknesses of the FM layer, Ta and its oxide (Ta_2_O_5_) were accurately determined by simulating the experimentally observed X-ray reflectivity (XRR) spectra (Table 1).

The deposited CFA films were investigated by glancing angle X-ray diffraction (GAXRD). To ascertain the film morphology, topographical scanning was performed using the Bruker make atomic force microscope (AFM) (Model - Dimension ICON *Scan* Assist). The film thickness and interface roughness were investigated by simulating the specular XRR spectra using the PANalytical X’Pert Reflectivity software (ver. 1.2 with segmented fit). To determine the chemical state (metal and/or oxide) of the top Ta as well as of the underlying FM layer, the binding energy shift employing X-ray Photoelectron Spectroscopic (XPS) measurements was carefully investigated. The XPS spectra on Si(100)/CFA/Ta bilayers were recorded by using a *SPECS* make system with an Al-K_*α*_ x-ray source (1486.6 eV) and a hemispherical energy analyzer with pass energy of 40 eV and a resolution of ~0.7 eV. The saturation magnetization was measured using the vibrating sample magnetometer (VSM) module of the Quantum Design make Physical Property Measurement System (QD PPMS-VSM) Evercool-II. The magnetic anisotropy behaviour was investigated by recording hysteresis loops at various azimuthal orientations by employing a homebuilt Magneto-Optical Kerr Effect (MOKE) set up in longitudinal geometry. A broadband lock-in amplifier (SR830 from Stanford Research Systems) detection based custom built ferromagnetic resonance (FMR) technique employing a vector network analyzer (Model 8719ES from HP) and a coplanar wave guide (see ref. 17 for details) was used to record the FMR spectra in the in-plane magnetic field sweep configuration while maintaining a constant microwave frequency in the 6–12 GHz range at 0 dBm power. The Helmholtz coils were used to modulate the dc-magnetic field at 211.5 Hz frequency using an excitation field amplitude of 1.3Oe.

## Results

**Figure 1:**
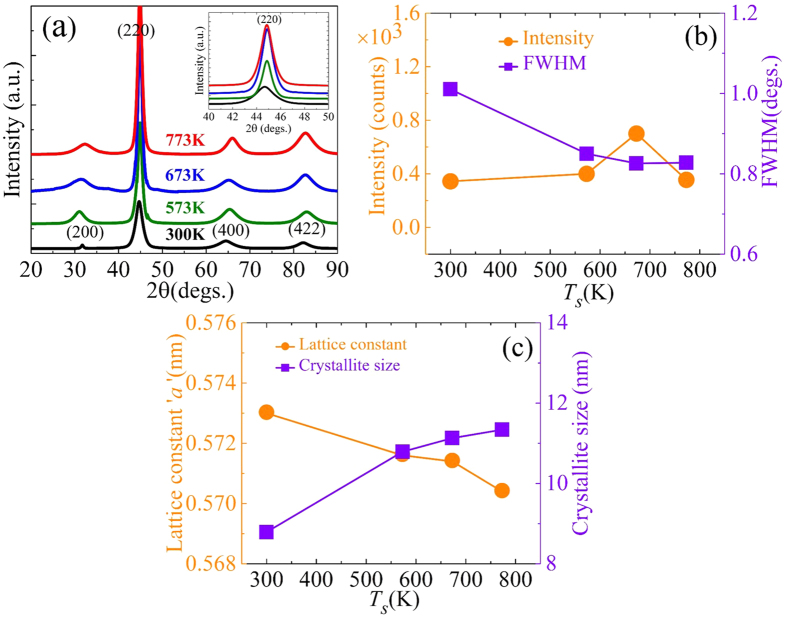
(**a**) XRD spectra for Si/CFA/Ta bilayers deposited at different *T*_*s*_ (inset shows the zoomed version of the (220) peak). (**b**) Variation of the intensity and FWHM of the (220)-peak with *T*_*s*. _(**c**) Variation of lattice constant and crystallite size inferred from the (220) peak with *T*_*s*_.

Figure 1(a) shows the XRD patterns of 53-nm-thick CFA Heusler alloy thin films grown at different *T*_*s*_. Whereas in the fully ordered *L2*_*1*_ structure, two diffraction peaks corresponding to (111) and (311) are expected to be present in the diffraction pattern^11^, the presence of the (200) diffraction peak may be taken as a clear sign of the *B2* phase. The presence of the (200) peak in our films (Fig. 1(a)) shows the formation of *B2* ordered CFA structure at all *T*_*s*_. As inferred from the graph, one can clearly see the additional presence of (220), (400) and (422) diffraction peaks indicative of the polycrystalline nature of the CFA thin films. Figure 1(b) shows the intensity and full width at half maximum (FWHM) of the (220) diffraction peak as a function of *T*_*s*_. The observed intensity of the (220) diffraction peak exhibits an increase at *T*_*s*_ = 673K owing to the improved crystalline quality. Moreover, in accordance with the enhancement in peak intensity, a corresponding minimum in FWHM is also observed at *T*_*s*_ = 673 K, indicating a transition from short-range to long-range crystallographic order^18^. The larger FWHM for the RT deposited film is due to the growth induced micro-strains^19^, and the decrease in the FWHM with the increase in *T*_*s*_ is the result of strain relaxation at higher *T*_*s*_. The lattice constant (*a*) evaluated using the (220) peak is found to decrease with *T*_*s*_. The values of *a* are in good agreement with values reported for the bulk *L2*_*1*_ phase (0.574 nm)^19^. The slight difference observed is due to the lattice misfit (−5.2%) between the Si-substrate (0.543 nm) and the CFA film (bulk value ~ 0.574 nm). In order to have further insight into the crystalline structure of the films, the crystallite sizes obtained from the (220) peak are shown in Fig. 1(c) as a function of *T*_*s*_. It is inferred from the graph that the crystallite size increases with increasing growth temperature. It is easily understandable considering the diffusion process from an atomic perspective. With increasing *T*_*s*_, the ad-atoms gain sufficient surface mobility which helps them to adhere to the nucleated islands thereby increasing the crystallite size^20^. It may be stressed here that clear distinction between the *L2*_*1*_ and *B2* CFA phases by XRD is difficult, and requires other probes such as neutron diffraction, NMR, etc. However, there exists an indirect approach to distinguish among the *L2*_*1*_, *B2* and *A2* phases of CFA. This essentially relies upon the density of states (DOS) based correspondence between the smallest *α* and the extent of structural ordering. The lowest damping corresponds to the perfectly ordered half-metallic *L2*_*1*_ phase, and highest damping corresponds to the disordered phase^21^. We will later return to this when the results of FMR measurements for all samples are presented.

**Figure 2:**
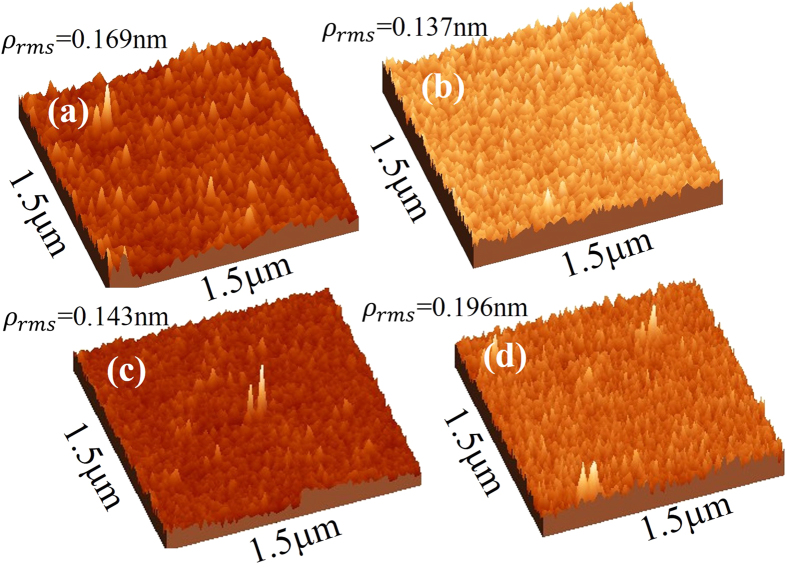
Topographical images of bilayer Si/CFA/Ta deposited at different *T*_*s*_; (**a**) 300 K, (**b**) 573 K, (**c**) 673 and (**d**) 773 K.

**Figure 3:**
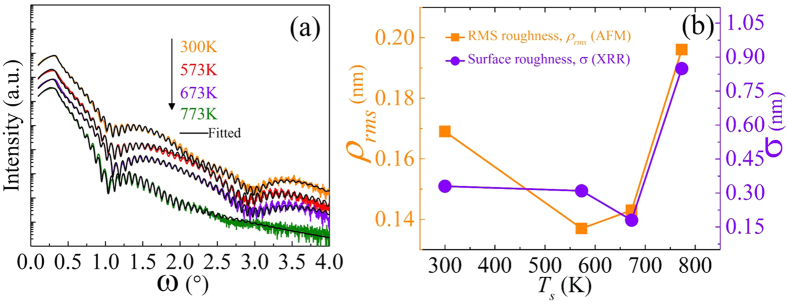
(**a**) X-ray reflectivity spectra recorded for bilayer Si/Co_2_FeAl/Ta thin films deposited at various *T*_*s*_ (colored lines represent the recorded curves, and black lines represent the simulated curves). (**b**) Effect of *T*_*s*_ on the surface roughness determined from AFM and XRR measurements (lines serve as guide to the eye).

The *T*_*s*_ dependent changes in the surface roughness of the Co_2_FeAl/Ta thin films have also been investigated using AFM imaging. The topological images recorded on these films are shown in Fig. 2. The observed root mean square roughness in all the samples is plotted in Fig. 3(b) as a function of *T*_*s*_. On increasing the *T*_*s*_ to 573 K the surface of the CFA film becomes relatively smoother as compared to the film deposited at RT. An optimally flat surface roughness of 0.137 nm is obtained at 573 K. Further increase of *T*_*s*_ up to 773 K resulted in significant increase in the film roughness, which could be attributed to the coalescence of relatively large size crystallites occurring at higher growth temperature^22^.

In order to accurately estimate the thickness, density, and the amount of interface diffusion (roughness) of individual layers in the Si(100)/CFA/Ta samples, specular XRR spectra were recorded. Figure 3(a) shows the XRR spectra (colored lines) recorded for all films grown at various *T*_*s*_ together with simulated spectra (black line) assuming a tri-layer model CFA/Ta/Ta_2_O_5_. The simulated XRR spectra can be seen to overlap remarkably well with the experimental data (Fig. 3(a)). The estimated values of film thickness, film density and associated surface/interface roughness are presented in Table 1. Distinct Kiessig fringes observed over the whole scanning range (~4°) for the films deposited at 300 K, 573 K and 673 K provide a strong evidence of sharp interfaces and excellent surface quality. However, in case of the film deposited at the highest *T*_*s*_ of 773 K, the fringes disappear above 3° which indicating increased interface roughness at the highest growth temperature. This is consistent with the AFM observations. The reduction in surface roughness of CFA/Ta thin films with an increase in *T*_*s*_ in the RT - 673 K range is associated with the increase in the ad-atom mobility and strain relaxation. The increase in roughness for 773 K deposited film is due to the high temperature induced inter-grain agglomeration in the films.

The density of the RT grown CFA layer is found to be 6.12 ± 0.06 g/cc, which increased up to 6.51 ± 0.06 g/cc as *T*_*S*_ increased to 673 K. The density decreases down to 6.34 ± 0.06 g/cc on further increase of the growth temperature to 773 K. This *T*_*s*_ dependent variation in film-density can be attributed to the *T*_*s*_ induced changes in the ad-atom mobility as explained above. For comparison, the surface roughness measured using AFM and XRR is shown in Fig. 3(b) as a function of *T*_*s*_. A thin layer of 2.2 nm of Ta_2_O_5_ was found to be formed on the top of the Ta layer due to the surface oxidation. As XPS is a powerful spectroscopic technique to examine the electronic properties such as electronic environment, oxidation state, multiplicity etc. of the various possible species formed near the surface of the films, in the next paragraph we present and analyse the XPS data recorded on the CFA/Ta bilayer sample deposited at *T*_*s*_ ~ 573 K.

**Figure 4:**
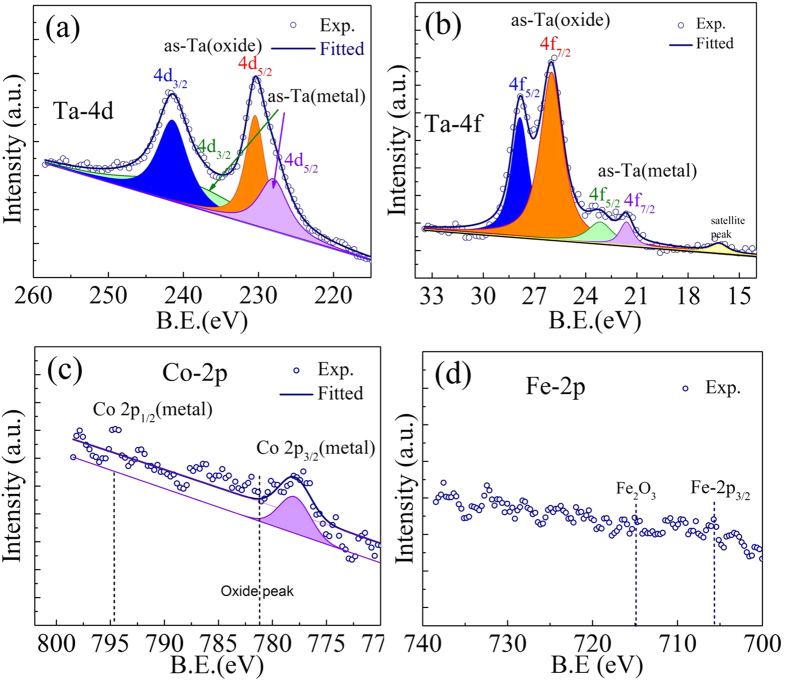
XPS spectra corresponding to (**a**) Ta**-4d, (**b**) Ta**-4f (**c**) Co-2p and (**d**) Fe-2p electronic levels recorded on Si/CFA (53 nm)/Ta(2 nm) thin film deposited at *T*_*s*_ ~ 573 K.

Figure 4 presents the XPS spectra recorded on the CFA/Ta thin film sample deposited at *T*_*s*_ ~ 573 K. The portion of recorded spectra corresponding to the binding energies of Ta, Co and Fe were de-convoluted in to their component metal and oxide-peaks and fitted by using the XPSPEAK 4.1 software which automatically takes care of the background during fitting using background correction tool. All spectra were calibrated using the C-1s peak (284.6 eV). The XPS investigation revealed the presence of Co, Fe, Ta and Ta_2_O_5_ in the Si(100)/CFA/Ta thin film. Whereas the select regions (in orange and blue) in Fig. 4(a) centered respectively at 230.4 eV and 241.6 eV binding energies are found to be distinctly associated with the different oxidation states of Ta *viz.*, 4d_5/2_ and 4d_3/2_, respectively corresponding to the presence of Ta_2_O_5_, the binding energy regions plotted in green and violet evidence the existence of the Ta-metal layer. Similarly in Fig. 4(b), the peaks at 26.0 eV and 27.8 eV symbolize the 4f_7/2_ and 4f_5/2_ electronic states, respectively corresponding to the formation of Ta_2_O_5_. Likewise, the corresponding Ta-metal peaks are represented for clarity in green and violet colors. The relatively lower intensity in the Ta-metal peaks compared to that of Ta_2_O_5_ (Fig. 4(a,b)) is consistent with the fact that the latter has passivated the underlying Ta which is relatively farther from the surface of the sample validating the layered structure modeled in the simulation of X-ray reflectivity data (Fig. 3 (a)). Figure 4(c,d) show the portions of XPS spectra corresponding to the Co-2p and Fe-2p peaks. While the XPS peaks corresponding to the binding energies of Co and Fe are not clearly resolved due to the fact that CoFe_2_Al lies quite far from the film surface, Co can be seen to be present in the metallic state with no signal seen corresponding to its oxides (expected at BE values marked by dotted lines for the Co-2p and Fe-2p levels). Thus, the XPS data provide further experimental support to the formation of a thin passivating layer of Ta_2_O_5_ over the Ta and underlying CFA as revealed from the XRR simulations. Zhu *et al*.^23^ and Liu *et al*.^24^ also reported that a 1 nm thin Ta metal layer is sufficient to ensure the formation of a passivating Ta_2_O_5_ layer for protecting the underlying Ta and FM layers.

**Figure 5:**
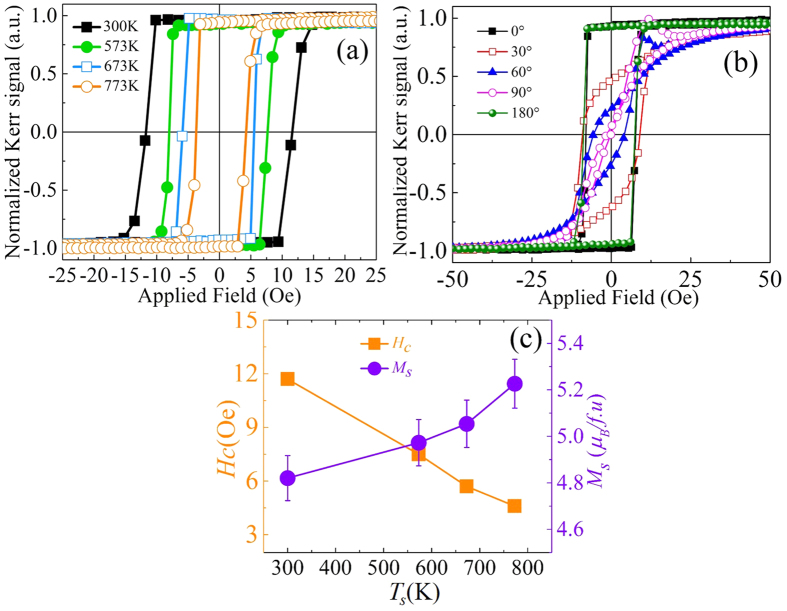
(**a**) MOKE hysteresis loops recorded at room temperature along the easy axis of magnetization for the CFA films deposited at different *T*_*s*_. (**b**) Angle dependent MOKE hysteresis curves recorded at different azimuthal angles on the Si/CFA (53 nm)/Ta (2 nm) thin film deposited at *T*_*s*_ ~ 573 K. (**c**) Dependence of *H*_*c*_ and *M*_*s*_on *T*_*s*_ (lines serve as guide to the eye).

In order to study the static magnetization response of these CFA films, magnetization hysteresis loops were recorded employing angle dependent MOKE in longitudinal geometry at room temperature. The hysteresis loops were recorded at different azimuthal angles in the range of 0–360°. The measured loops recorded along the easy axis of the CFA/Ta thin films grown at various *T*_*s*_ are shown in Fig. 5(a). All hysteresis loops exhibit 100% remenance (*M*_*r*_/*M*_*s*_ = 1) indicating nearly defect free samples and domain wall nucleation controlled switching of the magnetization. Figure 5(b) displays the hysteresis loops recorded at various azimuthal angles for the CFA/Ta film sample deposited at *T*_*s*_ = 573 K, which endorses the existence of uniaxial anisotropy in these CFA thin films. The origin of the uniaxial anisotropy could comprise of several sources, such as strain due to film-substrate lattice mismatch or the intrinsic magnetocrystalline anisotropy effect. It is noted that a characteristic spike is present near the positive coercive field in these hysteresis loops (Fig. 5(b)). It was reported by Muduli *et al*.^25^ that such spikes which are not visible in SQUID/VSM MH loops occur only in MOKE MH loops due to either the presence of higher order spin–orbit interactions or due to local magnetic ordering effects within the lattice. The saturation magnetization was measured using a PPMS-VSM and the variation of the saturation magnetization (*M*_*s*_) and coercivity (*H*_*c*_) evaluated from MOKE as function of *T*_*s*_ are shown in Fig. 5(c). The observed *M*_*s*_ values are very much comparable to the reported bulk value (5.0 μ_*B*_*/f.u*.)^26^. The observed variation in *M*_*s*_ indicates the improvement of local magnetic ordering of these thin films with the increase in *T*_*s*_^27^. The structural analysis discussed in the previous section suggested the improvement in the structural ordering with *T*_*s*_, which also improves the local magnetic ordering within the lattice of the Heusler alloy. Figure 5(c) shows that *H*_*c*_ decreases as the growth temperature *T*_*s*_ is increased. It may be pointed out that the *H*_*c*_ is influenced both by the pinning of the domain walls at the pinning centres/defects (such as concentration of grain boundaries, which varies inversely with the crystallite/grain size present in the films) as well as by the local variations in the magnetic anisotropy, *viz*., on the angle between the easy-axes of the domains with respect to direction of the applied magnetic field. Observation of higher (lower) *H*_*c*_ in room (high) temperature deposited films is attributed to the presence of relatively higher (lower) structural and magnetic disorder and smaller (larger) grain size of those films. This finds support from the observed increase in *M*_*s*_ (Fig. 5(c)) and the decrease in the number of grain boundaries (Fig. 1(c)) with increasing *T*_*s*_. It is expected that with lower defect density and enhanced crystalline quality at higher *T*_*s*_, a well-defined magnetocrystalline anisotropy eventually develops contributing little to the observed variation of *H*_*c*_.

We now turn to the *T*_*s*_-dependence of dynamic magnetization response of these films. To extract the line shape parameters, i.e., resonant field *H*_*r*_ and linewidth Δ*H*, the FMR spectra were fitted with the sum of the derivative symmetric and anti-symmetric Lorentzian functions^28^ as given by


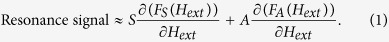


**Figure 6:**
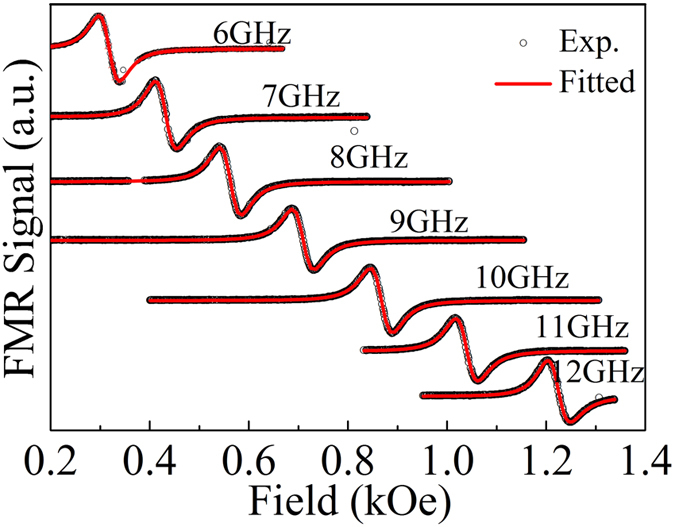
Frequency dependence of FMR spectra for the 573 K deposited CFA film (symbols represent the experimental data, and the solid line shows the fit using Equation (1)).

**Figure 7:**
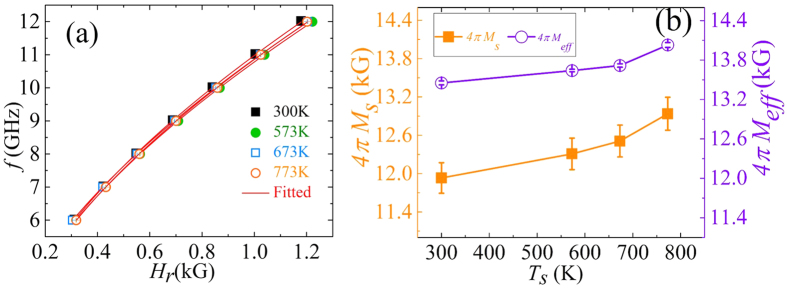
(**a**) *f* vs. *H*_*r*_ plot for the different films (symbols represent the experimental data, and the solid line shows the fit using Equation (2)). (**b**) *T*_*s*_ dependence of *4*π*M*_*eff*_ (extracted from the FMR results) together with *4*π*M*_*s*_ vs. *T*_*s*_ (lines serve as guide to the eye).

Here, *F*_*S*_(*H*_*ext*_) = Δ*H*^*2*^/{Δ*H*^*2*^ + (*H*_*ext*_−*H*_*r*_)^2^} and *F*_*A*_(*H*_*ext*_) = Δ*H*(*H*_*ext*_ − *H*_*r*_)/{Δ*H*^*2*^ + (*H*_*ext*_ − *H*_*r*_)^2^} are symmetric and antisymmetric Lorentzian functions, respectively, with *S* and *A* being the corresponding coefficients. The experimental and fitted FMR spectra of the film deposited at 573 K are shown in Fig. 6. Figure 7(a) presents the dependence of the extracted values of *H*_*r*_ on the microwave frequency.

The relationship between the resonance field *H*_*r*_ and the frequency *f* can be expressed by the famous Kittel’s formula^29^


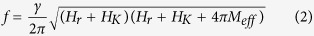


where γ is the gyromagnetic ratio (*γ* = *gμ*_*B*_/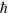) with *g* being the Lande’s splitting factor and taken as 2, 4*πM*_*eff*_ is the effective magnetization, and *H*_*K*_ is the in-plane uniaxial anisotropy field (the films possess uniaxial anisotropy as illustrated in Fig. 5(b)). The *H*_*r*_ vs. *f* data was fitted using Equation (2) to extract the effective magnetization, which is found to vary from 13.4 kG to 14.0 kG with increase in *T*_*s*_. The slightly lower experimental values of *4πM*_*s*_ (c.f. Fig. 7(b)) might be due to the error associated with the difficulty in accurately determining the volume of the sample for calculation of *4πM*_*s*_. The anisotropy field *H*_*K*_ is also determined from the fitting of the *H*_*r*_ vs. *f* curve and its value is found to lie in the range of 1–25 Oe depending upon the value of *T*_*s*_.

**Figure 8:**
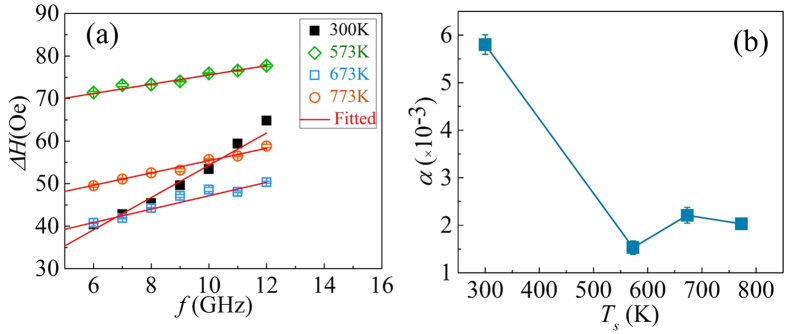
(**a**) Δ*H* vs. *f* plots for CFA films grown at different *T*_*s*_ (symbols represent the experimental data, and the solid line shows the fit using Equation (3)). (**b**) Plot of Gilbert damping constant *α* vs. *T*_*s*_ (lines serve as guide to the eye).

The frequency dependence of the linewidth Δ*H* vs. *f* is shown in Fig. 8(a). This data is used to obtain information about the intrinsic Gilbert damping constant and the extent of extrinsic and local magnetic relaxation due to film inhomogeneity in the sample^30^. Quantitatively, Δ*H* (*f*) is expressed as


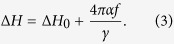


It is important to point out here that the first term Δ*H*_0_ is the frequency independent film inhomogeneity linewidth contribution, while the second term corresponds to the frequency dependent contribution associated with the intrinsic Gilbert relaxation.

Figure 8(a) shows the Δ*H vs*. *f* plots together with the fits obtained using Equation (3) to extract the values of *α* for the films deposited at different *T*_*s*_. The extracted values of the damping constant *α* for all the samples are plotted in Fig. 8(b). The lowest value of *α* is found to be 0.0015 ± 0.0001 for the film deposited at 573 K. This value competes excellently with the smallest *α*-values of 0.001 and 0.0011 reported by Mizukami *et al*.^31^ and Belmeguenai *et al*.^32^, respectively. It may, however, be pointed out that in these reports^31^,^32^, the CFA thin films were deposited on MgO substrates and also required an additional process step of post-annealing at 600 °C. Here, we would like to mention the advantage of the ion beam sputtering (IBS) technique (employed in the present work) owing to the higher energy (~20 eV) of the sputtered species as compared to the DC sputtering technique (~2–4 eV). The additional energy given to the species helps during the nucleation and growth stages of the deposition process to form films with minimal defect density corresponding to the lowest free-energy. Thus, the CFA films studied in the present case have been obtained for as-prepared CFA films (without post annealing) using the energy enhanced IBS technique. In our opinion, the extremely small value of *α* obtained in these CFA films is correlated with improved structural quality (evident from the observation of the (220) reflection, Fig. 1(a), crystallization of the *B2* ordered phase (evident from the presence of the (200) reflection, Fig. 1(a), higher value of *4πM*_*s*_ (Fig. 7(b)), low pinning/coercivity (Fig. 5(c)), and extremely low interface/surface roughness ~ (sub-atomic thickness as revealed by the XRR/AFM results (Fig. 3(b)). The inhomogeneous linewidth broadening extracted from the fitting is found to be 16.4 ± 2.5 Oe and 64.6 ± 0.9 Oe for room temperature and 573 K temperature deposited films, respectively. The observed inhomogeneous linewidth broadening did not vary systematically with *T*_*s*_, rather it is found to be smallest for the RT-grown film and largest for the film deposited at 573 K. There are several reasons for the linewidth broadening, for example two-magnon scattering (TMS) and film inhomogeneities^33^,^34^.

To rule out the possibility of a TMS contribution in our film samples, we have performed a detailed analysis including the TMS contribution as described in the theory of two-magnon scattering by Arias and Mills^35^. The linewidth has three main contributions: (i) Δ*H*_*inh*_; inhomogeneity of the sample which is independent of frequency, (ii) Δ*H*_*G*_; Gilbert damping term which is intrinsic in nature and dependent on frequency, and (iii) Δ*H*_2*mag*_; TMS term which is extrinsic in nature and frequency dependent. Thus the overall linewidth of the system can be written as


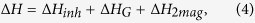


where 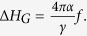
The linewidth due to TMS is given by


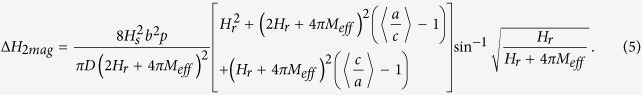


To extract the Gilbert damping constant considering the TMS contribution to the FMR linewidth, Δ*H* vs. *H*_*r*_ plots were fitted using Equations (4 and 5). For this, the term 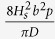 is treated as a parameter *K*, and the parameters *b* (height/depth of bump/pit defects), *p* (fraction of surface covered by defects), *D* (exchange stiffness), and *H*_*s*_ (surface anisotropy field << 4π*M_eff_*) are assumed to be constants for the material under study (for details see ref. 35). It may be noted that *a* and *c* represent the in-plane dimensions of the defects which are considered as rectangular in shape for cubic crystals, and the angular brackets are used to denote average values of the defect dimensions.

**Figure 9:**
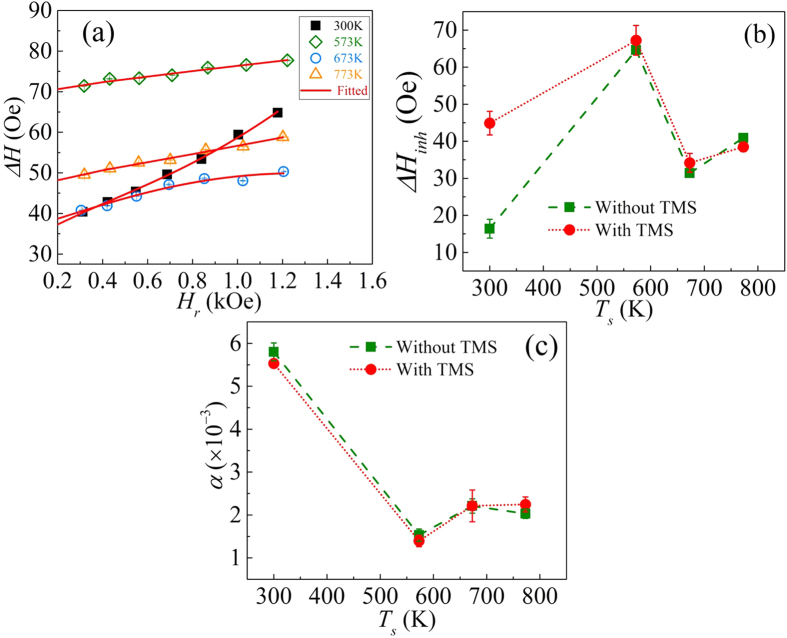
(**a**) The observed line broadening Δ*H* versus resonance field *H*_*r*_ (symbols) of the films sputtered at different *T*_*s*_, and fit (solid line) considering the TMS contribution described by Equations (4 and 5). Fig. (**b**,**c**) show the dependency of Δ*H*_*inh*_ and Gilbert damping constant *α* on *T*_*s*_ with and without considering the TMS contribution.

Figure 9(a) shows the Δ*H* vs. *H*_*r*_ data fitted using Equations (4 and 5); the red lines are the fitted results. The *H*_*k*_ and *M*_*eff*_ values were kept fixed during the fitting; their values were obtained using Kittel’s equation as shown in Fig. 7(a). The observed values of the fitting parameters *K*, *α*, 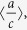 and Δ*H*_*inh*_ are presented in Table 2. The extracted values of the inhomogeneous linewidth Δ*H*_*inh*_ and Gilbert damping constant α, with and without incorporation of TMS in the expression for the FMR linewidth, are plotted in Fig. 9(b,c), respectively. The extracted values of Δ*H*_*inh*_ and *α* using Equation (4), considering TMS, are nearly equal to the values obtained using Equation (3), without considering TMS. This can also be inferred from the average values of the defect topology ratio 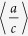 which are found to be nearly constant~0.8–0.9 for all the samples. It may here be pointed out that the thickness of the samples in the present study (53 nm) is very large as compared to the thicknesses (~few nm) assumed in the TMS model given by Arias and Mills^35^. Thus in the 53 nm thick samples, the surface anisotropy is negligible. Evidently, the linear dependency of the linewidth throughout the applied frequency range provides direct indication of the negligible contribution of TMS^36^ and therefore it may be ignored in the present analysis. As a result the observed linewidth broadening is attributed to the film inhomogeneities. This involves a number of microstructural factors, e.g., grain-size effects, random/local variations in the magnetocrystalline anisotropy and anisotropy due to shape/local strain among the grains in the polycrystalline films, each of which could result in local resonance effects^37^,^38^,^39^. The non-systematic variation observed in Δ*H*_0_ will be addressed in a forthcoming study, where in out-of-plane FMR measurements will be performed thereby avoiding the two-magnon contribution to the linewidth. Epitaxial films invariably show smaller linewidth^18^. Nevertheless, the present work suggests that owing to the small value of the damping constant polycrystalline Co_2_FeAl electrodes can be used in magnetic tunnel junctions and spin transfer torque devices to improve device functionality/performance.

In summary, the growth of Co_2_FeAl Heusler alloy thin films at different substrate temperatures on Si(100) by employing ion-beam sputtering deposition technique is reported. Based on the detailed investigations of the microstructure, surface morphology, X-ray reflectivity, and the static and dynamic magnetization properties, it is found that these CFA thin films exhibit the *B2* ordered phase and possess improved crystallinity, very low surface roughness (<3 Å), and minimal anti-site disorder consistent with the observed higher saturation magnetization. Most importantly, a record low Gilbert damping constant of 0.0015 ± 0.0001 is obtained corresponding to the optimum substrate temperature of ~573 K. This lowest value of the damping constant observed in the CFA films deposited on silicon substrate compares excellently well with values reported for MBE grown CFA films deposited on single crystalline MgO substrates. The study clearly suggests that thermally stable half-metallic ferromagnetic Co_2_FeAl thin films sputtered on the industrially popular Si substrate have remarkable application potential owing to their very low magnetic damping for developing novel spin transfer torque devices.

## Additional Information

**How to cite this article**: Husain, S. *et al*. Growth of Co_2_FeAl Heusler alloy thin films on Si(100) having very small Gilbert damping by Ion beam sputtering. *Sci. Rep*. **6**, 28692; doi: 10.1038/srep28692 (2016).

## Figures and Tables

**Table 1:** 

*T*_*s*_ (K)	300	573	673	773
Layer	ρ(g/cc) ± 0.06, *t*(nm) ± 0.01, *σ*(nm) ± 0.03	ρ(g/cc) ± 0.06, *t*(nm) ± 0.01, *σ*(nm) ± 0.03	ρ(g/cc) ± 0.06, *t*(nm) ± 0.01, *σ*(nm) ± 0.03	ρ(g/cc) ± 0.06, *t*(nm) ± 0.01, *σ*(nm) ± 0.03
Si	3.26 60000 0.41	3.26 60000 0.37	3.26 60000 0.33	3.26 60000 0.57
CFA	6.12 53.17 0.41	6.19 52.45 0.60	6.51 52.83 0.78	6.34 52.21 1.23
Ta	15.80 1.92 0.19	14.15 1.85 0.24	15.67 1.69 0.33	14.41 1.81 0.33
Ta_2_O_5_	7.05 2.26 0.32	7.93 2.34 0.31	7.85 2.26 0.18	7.40 2.52 0.85

The XRR simulated parameters i.e., ρ, *t*, and σ for the bilayer thin films [Si/CFA(53)/Ta(2)] deposited at different *T*_*s*_. Here ρ, *t*, and σ respectively refer to the density, thickness, and interface width of the individual layers.

**Table 2:** 

T_s_	Damping (α)	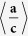	*K*	Δ *H*_*inh*_	4π*M*_*eff*_
(K)			(G^−1^)	(Oe)	(kG)
300	0.00553	0.8442	0.78493	44.9	13.45
	(±1.00798 × 10^−4^)	(±0.00595)	(±0.03938)	(±3.17732)	(±0.00303)
573	0.00139	0.94008	0.17882	67.3	13.64
	(±1.32101 × 10^−4^)	(±0.0191)	(±0.05728)	(±4.01)	(±0.019)
673	0.00221	0.94746	0.85334	34.2	13.72
	(±3.70198 × 10^−4^)	(±0.00461)	(±0.55447)	(±2.52)	(±0.026)
773	0.00224	0.94552	0.46399	38.5	14.03
	(±1.73281 × 10^−4^)	(±0.00469)	(±0.28994)	(±1.24)	(±0.036)

The parameter 4π*M*_*eff*_ is obtained by fitting of *H* vs. *f* (Fig. 7) using Kittel’s equation (2).
